# Chromosomal aberrations in human hepatocellular carcinomas associated with hepatitis C virus infection detected by comparative genomic hybridization

**DOI:** 10.1038/sj.bjc.6690638

**Published:** 1999-08

**Authors:** C Sakakura, A Hagiwara, H Taniguchi, T Yamaguchi, H Yamagishi, T Takahashi, K Koyama, Y Nakamura, T Abe, J Inazawa

**Affiliations:** First Department of Surgery, Department of Hygiene, Kyoto Prefectural University of Medicine, Kamigyo-ku, Kawaramachi-dori, Kyoto, 602, Japan; Human Genome Center, Institute of Medical Science, Tokyo University, 4-6-1 Shirokanedai, Minato-ku, Tokyo, 108-8639, Japan; Department of Molecular Cytogenetics, Medical Research Institute, Tokyo Medical and Dental University, 1-5-45 Yushima, Bunkyo-ku, Tokyo, 113-8519, Japan

**Keywords:** hepatocellular carcinomas, CGH, chromosomal aberrations

## Abstract

Thirty-five hepatocellular carcinomas (HCCs) associated with hepatitis C virus (HCV) were analysed by comparative genomic hybridization (CGH), to screen for changes in copy-number of DNA sequences. Chromosomal losses were noted in 1p34–36 (37%), 4q12–21 (48%), 5q13–21 (35%), 6q13–16 (23%), 8p21–23 (28%), 13q (20%), 16q (33%) and 17p13 (37%). Gains were noted in 1q (46%), 6p (20%), 8q21–24 (31%) and 17q (43%). High level gains indicative of gene amplifications were found in 7q31 (3%), 11q13 (3%), 14q12 (6%) and 17q12 (3%); amplification at 14q12 may be characteristic for HCCs. No significant difference in chromosomal aberrations was noted between carcinomas associated with HCV-infection in our study and those reported earlier in HCCs infected with hepatitis B virus (HBV), indicating that both HBV- and HCV-related carcinomas may progress through a similar cascade of molecular events. © 1999 Cancer Research Campaign


*British Journal of Cancer* (1999) **80**(12), 2034–2039
© 1999 Cancer Research Campaign
Article no. bjoc.1999.0638

### Chromosomal aberrations in human hepatocellular carcinomas associated with hepatitis C virus infection detected by comparative genomic hybridization

**C Sakakura,[1,2] A Hagiwara,[1] H Taniguchi,[1] T Yamaguchi,[1] H Yamagishi,[1] T Takahashi,[1] K Koyama,[3] Y Nakamura,[3] T Abe[2] and J Inazawa[2,3,4]**

[1]First Department of Surgery, [2]Department of Hygiene, Kyoto Prefectural University of Medicine, Kamigyo-ku, Kawaramachi-dori, Kyoto 602, Japan; [3]Human Genome Center, Institute of Medical Science, Tokyo University, 4-6-1 Shirokanedai, Minato-ku, Tokyo 108-8639, Japan; [4]Department of Molecular Cytogenetics, Medical Research Institute, Tokyo Medical and Dental University, 1-5-45 Yushima, Bunkyo-ku, Tokyo 113-8519, Japan

**Summary** Thirty-five hepatocellular carcinomas (HCCs) associated with hepatitis C virus (HCV) were analysed by comparative genomic hybridization (CGH), to screen for changes in copy-number of DNA sequences. Chromosomal losses were noted in 1p34–36 (37%), 4q12–21 (48%), 5q13–21 (35%), 6q13–16 (23%), 8p21–23 (28%), 13q (20%), 16q (33%) and 17p13 (37%). Gains were noted in 1q (46%), 6p (20%), 8q21–24 (31%) and 17q (43%). High level gains indicative of gene amplifications were found in 7q31 (3%), 11q13 (3%), 14q12 (6%) and 17q12 (3%); amplification at 14q12 may be characteristic for HCCs. No significant difference in chromosomal aberrations was noted between carcinomas associated with HCV-infection in our study and those reported earlier in HCCs infected with hepatitis B virus (HBV), indicating that both HBV- and HCV-related carcinomas may progress through a similar cascade of molecular events.

**Keywords:** hepatocellular carcinomas; CGH; chromosomal aberrations

Hepatocellular carcinoma (HCC) is one of the most frequent human cancers worldwide, and it carries a very poor prognosis (Whelan et al, 1993). Although genetic changes underlying the development and progression of HCC are poorly understood, there exist some well-known predisposing factors such as persistent hepatitis due to viral infection and exposure to mycotoxins (Rensburg et al, 1985; Nalpas et al, 1991; Tanaka et al, 1991). In fact, most of HCC are associated with a background of chronic liver disease (chronic viral hepatitis or cirrhosis). Both activation of cellular oncogenes and inactivation of tumour suppressor genes have been implicated in previous studies (Rogler et al, 1992; Tabor et al, 1994; Di Bisceglie et al, 1997; Nishida et al, 1997).

Cellular proto-oncogenes may be activated through insertion of a viral genome, a process similar to integration of retrovirus; in a few cases, mutagenesis resulting from integration of hepatitis B virus (HBV) into cellular genes has appeared to be linked to hepatocarcinogenesis (Wang et al, 1990). However, since most HCCs accompanied by infection with HBV contain viral DNA integrated in the genome, this mechanism, as well as oncogene activation, has been proposed as a general contributor to the development of hepatocellular carcinoma (Simon and Carr, 1995). By contrast, to our knowledge hepatitis C virus (HCV) is never integrated into the genomes of hepatocytes (Fong et al, 1991; Kasai et al, 1996; Di Bisceglie et al, 1997). The carcinogenic mechanisms of both viruses are still under intensive investigations.

*Received 27 October 1998*
*Accepted 16 February 1999*

*Correspondence to:* J Inazawa, Department of Molecular Cytogenetics, Medical Research Institute, Tokyo Medical and Dental University, Yushima 1-5-45, Bunkyo-ku, Tokyo 113-8519, Japan

Among the tumour suppressors most frequently inactivated in HCCs is the p53 gene, mutated in 25–60% of these tumours (Oda et al, 1992; Fujimoto et al, 1994). Losses of heterozygosity (LOH) at chromosomes 1p, 4q, 5q, 8p, 11p, 13q, 16q, 17p and/or 22q are common in HCC (Tsuda et al, 1990; Zhang et al, 1990; Fujimori et al, 1991; Simon et al, 1991). Classical cytogenetic studies have detected losses of the same chromosome arms in HCCs (Fourel et al, 1994). Therefore, some putative tumour suppressor genes at these loci may be involved in the development and progression of HCC.

Comparative genomic hybridization (CGH) is a molecular cytogenetic method that makes it possible to survey the entire genome for gains and losses of DNA sequences (Kallioniemi et al, 1992, 1994). The utility of CGH is based on the concept that regions with increased copy number reveal chromosomal sites that may contain dominant oncogenes, whereas regions with decreased copy number may be loci of putative tumour suppressor genes (Kallioniemi et al, 1992, 1994). CGH reliably screens the entire human genome, and therefore allows detection of any chromosomal sites that are likely to contain genes with an important role in tumour development (Ariyama et al, 1998; Sakakura et al, 1999).

In the study reported here, CGH analysis was performed in 35 cases of HCC with HCV infection (hereafter, HCV-HCC) in order to identify those regions that contain potential oncogenes or tumour suppressor genes responsible for hepatocellular carcinogenesis.

### MATERIALS AND METHODS

### Primary tumour specimens

The material consisted of 35 primary human hepatocellular carcinomas with HCV infection. The clinical stage distribution of these

**Table 1** DNA copy number changes in each of 35 hepatocellular carcinomas

| Sample no. | Gain | Loss | Histology, stage |
|---|---|---|---|
| 1 | – | 12q, X | Ed1, T2N0M0 Stage 2 |
| 2 | 1q, **7q31**, 12q, **14q12** | 1p | Ed3, T3N0M0 Stage 3 |
| 3 | **14q12**, 8q,9q,16p,19p,20 | 1p,4q,5q,8p,10,11q,13q | Ed3, T1N0M0 Stage 1 |
| 4 | 8q | 1p31–36, 4q, 6q, 8p, | Ed2, T2NXM0 Stage 2 |
| 5 | 1q,3,6,17q | 4,11,12p,13q,14q,15q,18p | Ed3, T3N0M0 Stage 3 |
| 6 | 1q,5p, 8q | 4q,5,8p,14q,17p,19 | Ed2, T2N0M0 Stage 2 |
| 7 | – | – | Ed2, T3N0M0 Stage 3 |
| 8 | 1q, 7,15q,16p | 1p,4q,5q,13q,16q | Ed2, T4N0M0 Stage 4a |
| 9 | 1q,8q,17q, 20 | 2,3,4,5q,6q 14–26,11 | Ed2, T2N0M0 Stage 2 |
| 10 | 1q,8q,10,14q | 1p,7q,10q,16,17,18q,19,20,21q | Ed2, T1N0M0 Stage 1 |
| 11 | 3,10q,12,17q,20 | 1p34–36,3p12–13,4q,7,16,17p13 | Ed2, T4N0M0 Stage 4a |
| 12 | 1q,3, 4p,5q,6,16,17q | 10,15q,21q,Xp,Xq12–21 | Ed4, T3N0M0 Stage 3 |
| 13 | 3,17q,Xq | 5p,5q11–32,15q,18 | Ed2, T1N0M0 Stage 1 |
| 14 | 7,8q,17q,19q,20,22q | 8p,12p | Ed3, T4NXM0 Stage 4a |
| 15 | 1q,6p,7p14–22,14q,15q,17q,21q | 3p22–26,4q,6q,7p,12q | Ed2, T2N0M0 Stage 2 |
| 16 | 1q,1q,8q,15q | 9q,12q24,16,17,19,20,21q | Ed2, T2N0M0 Stage 2 |
| 17 | 15q | 1p | Ed2, T3N0M0 Stage 3 |
| 18 | 1q,2,8q,11,12,14q,17q,18,19 | 1p,4q | Ed3, T3N0M0 Stage 3 |
| 19 | 1q,3,4p,11p,17,19q,21q | 1p,4q,6q,9q22–24,16,17p13,18,19 | Ed4, T3N0M0 Stage 3 |
| 20 | – | 17p | Ed2, T2N0M0 Stage 2 |
| 21 | 1q,2,3,**11q13**,17q,X, | 4q12–31,9q31–34,17p,19,21q,22q | Ed1, T3N0M0 Stage 3 |
| 22 | 2,4,11p,13q,15q,19 | 5q14–23,6q | Ed2, T3N0M0 Stage 3 |
| 23 | 6p,17q, | 6q,17p,8p21–23 | Ed2, T3N0M0 Stage 3 |
| 24 | 1q,8q,10p,17q,20,21q | 1p,2,4,5,8q,9p22–24,13q,16q,17p,X | Ed3, T2N0M0 Stage 2 |
| 25 | 2,6,16p,X | 1p,4q,13q,16q,18q | Ed2, T3N0M0 Stage 3 |
| 26 | 1q,2p,6p,8q,17,20,X | 1p,5q,6q,16,18,20 | Ed2, T4N0M0 Stage 4a |
| 27 | **17q12** | 17p,19,22q | Ed4, T4N0M0 Stage 4a |
| 28 | – | – | Ed2, T2N0M0 Stage 2 |
| 29 | – | – | Ed2, T3N0M0 Stage 3 |
| 30 | 8q,18,19q,X | 1q22–32,2q,4,6q,13q,17,19 | Ed2, T4N0M0 Stage 4a |
| 31 | 1q,4p | 4q,5q,8p,16,19,22q | Ed3, T2N0M0 Stage 2 |
| 32 | 2p,17,8q21–24 | 5q,6,8p,17p,21q | Ed2, T3N0M0 Stage 3 |
| 33 | 11p,16p,17q,19p,20 | 4,8p,9p,16q,19,21q | Ed3, T4N0M0 Stage 4a |
| 34 | 1q,11p,15q, 21q | 5q, 8p, 9,16q,17p13,19 | Ed2, T3N0M0 Stage 3 |
| 35 | 6p,9q,11, 12q14–22 | 1p, 4, 5q, 8p21–23,12p,13q,16q,17p | Ed3, T2N0M0 Stage 2 |

*The regions of high level gain are written in bold. TNM classification of UICC was used for staging system. Edmondson classification was used for histological classification.

cases was as follows: stage 1, three cases; stage 2, 11 cases; stage 3, 14 cases; stage 4, seven cases. High molecular weight tumour DNA was isolated from homogenized tumour specimens using standard protocols. Normal human male DNA was also isolated from the peripheral blood of a male volunteer as using a reference DNA for CGH.

### CGH and digital image analysis

CGH was performed using directly fluorochrome-conjugated DNA as described elsewhere (Ariyama et al, 1998; Sakakura et al, 1999). Three single-colour images (DAPI, Spectrum green and Texas red fluorescence) were collected from each metaphase spread using an epifluorescence microscope (Nikon, Tokyo, Japan), a cooled charge-coupled device (CCD) camera (Hamamatsu Photonics, Hamamatsu, Japan), and analysed using a digital image analysis system, The Power Gene, Mac Probe (PSI, Perceptive Scientific Instrument). Chromosomal regions where the mean ratio fell below 0.8 were therefore considered to reflect losses of DNA (underrepresentation), whereas regions where the mean ratio exceeded 1.2 were considered gained (overrepresentation) in the tumour genome. Overrepresentations were considered to be high-level amplifications when the fluorescence ratio exceeded 1.5 (Sakakura et al, 1999). Heterochromatic regions near the centromeres and the entire Y chromosome were excluded from analysis.

### RESULTS

An overview of genetic changes in 35 HCCs is shown in Figure 1 and listed in Table 1. Thirty-two tumours (92%) showed DNA sequence copy number changes, which was significantly higher than previous cytogenetic studies had indicated (Lowichik et al, 1996). This reflects the power of CGH in revealing aberrations across the genome in uncultured cells. Three tumours had no copy number changes. None of six normal liver tissues showed any gains or losses of DNA sequence copy number (data not shown). Losses in HCC predominated over gains with a ratio of 1:1.2. On average, there were 7.6 (range 0–12) aberrations per primary tumour: 3.4 gains (range 0–6) and 4.2 losses (range 0–7).

The most common copy number changes were losses at 1p34–36 (13/35; 37%), 4q12–21 (17/35; 48%), 5q13–21 (12/35; 35%), 6q13–16 (8/35; 23%), 8p21–23 (10/35; 28%), 13q (7/35; 20%), 16q (12/35; 33%) and 17p13 (13/35; 37%). Gains were seen in 1q (16/35; 46%), 6p (7/35; 20%), 8q21–24 (11/35; 31%) and 17q (15/35; 43%). Minimal overlapping regions of common DNA copy number changes in these HCCs, and their relationship to known locations of oncogenes, tumour suppressor genes, or

*© 1999 Cancer Research Campaign*

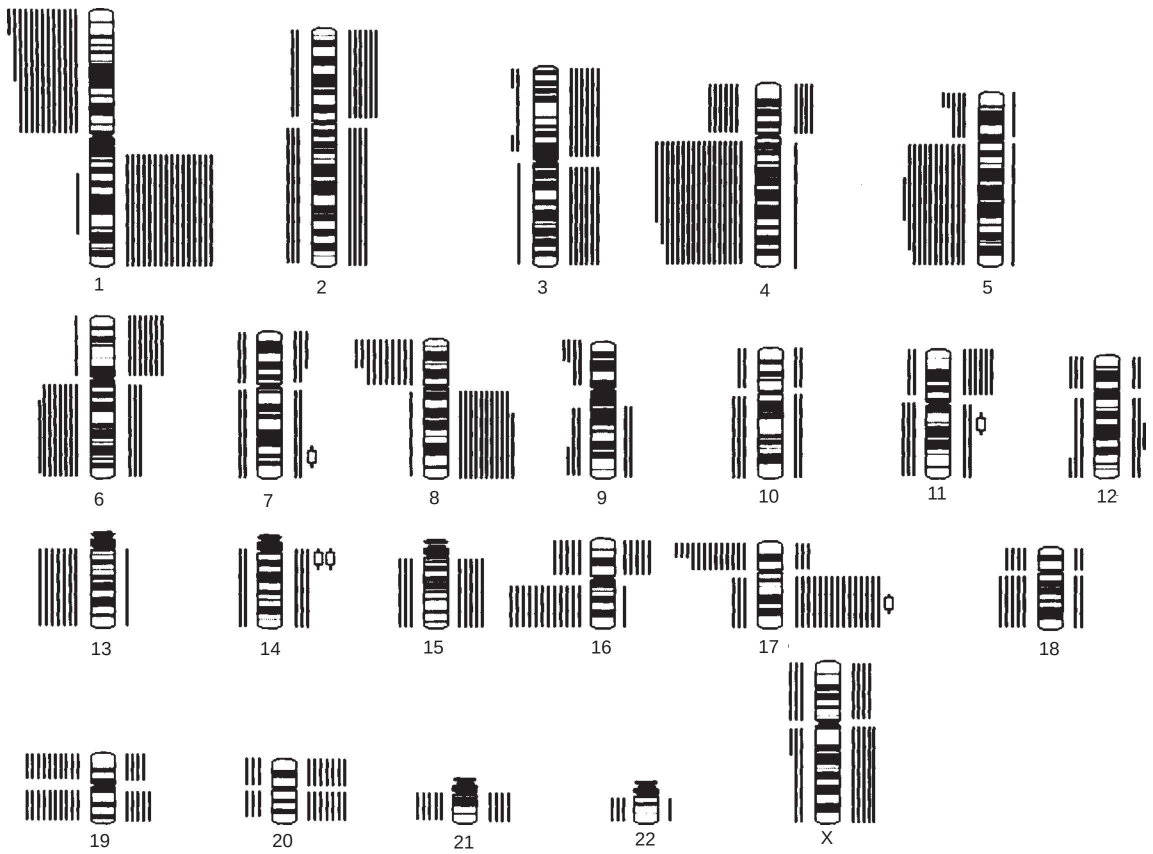

**Figure 1** Schematic view of DNA copy number changes in 35 human hepatocellular carcinomas, with CGH data plotted in ideogram form. The 22 autosomal chromosomes and the X chromosome are represented by ideograms showing G-banding patterns, oriented with p-arms at the top. Each region of decrease in DNA is represented as a thin solid line to the left of and parallel to the chromosomal region where it occurs, as judged by computerized green-to-red profiles (see Materials and Methods). Each copy number increase is likewise represented as a thin solid line, to the right of the region where it occurs. Regions of frequent copy number change have multiple parallel lines beside them, and gene amplifications are indicated by open rectangles. The data are summarized in Tables 1 and 2

adhesion molecule genes are listed in Table 2. High level gains indicative of amplified genes were found in 7q31 (1/35; 3%), 11q13 (1/35; 3%), 14q12 (2/35; 6%) and 17q12 (1/35; 3%).

Figure 2A shows the typical two-colour image among our panel of CGH. The DAPI-stained image of the same metaphase from which the chromosomes were identified is shown in Figure 2B. Its copy number profile is shown in Figure 2C. DNA gains are evident on chromosomes 1q, 5p and 8q. Losses on chromosomes 4, 5q, 8p, 14q 17p and 19 are also readily apparent.

### DISCUSSION

Screening for HBV prior to blood transfusion has decreased the incidence of HCC with HBV infection in Japan, and more than 80% of HCC in Japan is now associated with HCV infection (Tanaka et al, 1991; The Liver Cancer Study Group in Japan, 1994; Takano et al, 1995). The role of HCV-infection in the aetiology of HCC and cirrhosis now seems to be more important than chronic hepatitis B infection. Although HBV is randomly integrated into the genome of hepatocytes in more than 90% of HCC with HBV infection (HBV-HCC) (Tokino et al, 1987; Tabor et al, 1994), HCV does not integrate into the genome as far as we know (Fong et al, 1991; Di Biscegli et al, 1997). The mechanism by which HCV contributes to development of HCC is unclear. However, as patients with HCV infection tend to be older and show more severe liver cirrhosis than patients with HBV-HCC, not only the background but also the mechanism involved in initiation of carcinogenesis may be different for the two viruses.

**Table 2** Minimal overlapping regions of common DNA copy number changes in hepatocellular carcinoma

| DNA copy number | Locus | Frequency | Putative target genes |
|---|---|---|---|
| Increase | 1q | 46% (16/35) | *PTPRC, ARG* |
| | 6p | 20% (7/35) | *PIM1* |
| | 8q21–24 | 31% (11/35) | *MYC* |
| | 17q | 43% (15/35) | *ERBB2* |
| High level gain (amplification) | | | |
| | 7q31 | 3% (1/35) | *MET* |
| | 11q13 | 3% (1/35) | *HST1/INT2* |
| | 14q12 | 6% (2/35) | |
| | 17q12 | 3% (1/35) | *ERBB2* |
| Decrease | 1p34–36 | 37% (13/35) | *TP73* |
| | 4q12–21 | 48% (17/35) | |
| | 5q13–21 | 35% (12/35) | *APC* |
| | 6q13–16 | 23% (8/35) | |
| | 8p21–23 | 28% (10/35) | |
| | 13q | 20% (7/35) | *RB* |
| | 16q | 33% (12/35) | *E-Cadherin (CDH1)* |
| | 17q13 | 37% (13/35) | *TP53* |

© 1999 Cancer Research Campaign

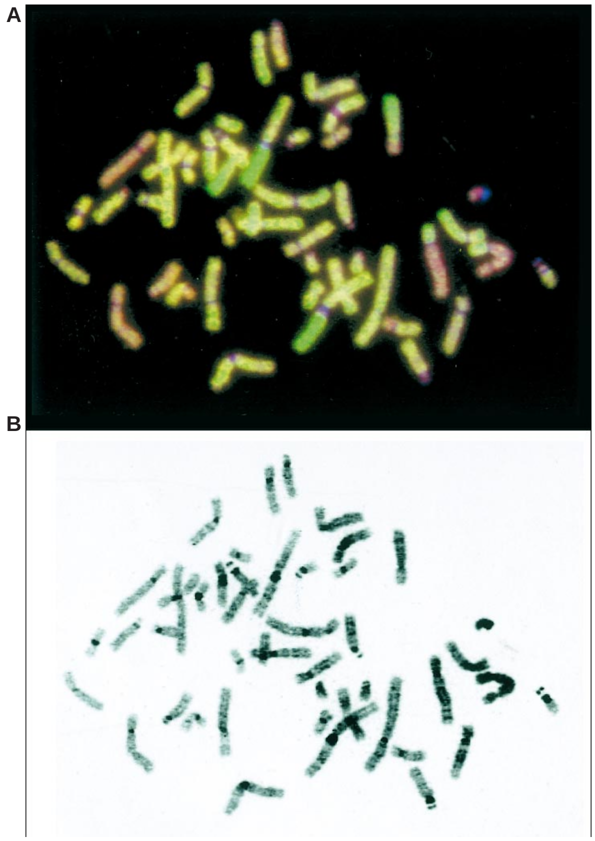

**Figure 2 (A)** Typical two-colour image of CGH in hepatocellular carcinoma. Differentially labelled tumour (green) and normal (red) DNAs were hybridized onto a normal human metaphase spread (46, XY). Chromosomal regions that are overrepresented in the tumour appear green, regions that are under-represented appear red and unrelated regions appear yellow. Chromosomes showing copy number changes are numbered at their q ends; DNA gains are evident on chromosomes 1q, 5p and 8q. Losses on chromosomes 4, 5q, 8p, 14q and 17p are also readily apparent. **(B)** DAPI-stained image of the same metaphase cell shown in panel **A**. DAPI banding patterns were used for chromosome identification.

Our CGH analysis of 35 HCV-HCCs showed frequent imbalances on chromosomes 1, 4q, 5q, 6, 8, 16q and 17. These data are concordant with the results recently reported for HBV-HCCs (Marchio et al, 1997); a similar cohort of oncogenes and suppressor genes at these loci may be involved in the development and progression of both HBV- and HCV-related HCC. Our CGH results also agree with those of investigators who found no correlation between LOH in specific chromosomal regions and chronic infections with hepatitis B or C (Konishi et al, 1993; Nagai et al, 1997).

LOH analyses have revealed that allelic loss of 4q is a frequent change in early-stage HCCs (Fujimoto et al, 1991; Nagai et al, 1996), and this chromosomal region is thought to harbour one or more tumour suppressor genes (Buetow et al, 1989; Yeh et al, 1996). To our knowledge, underrepresentation of this locus, as revealed by CGH, has never been reported in other types of cancers, so HCC-specific tumour suppressor genes may exist there. Another group of investigators reports that loss of 4q21–22 region occurred preferentially in well-differentiated HCC and they considered this a secondary event that increased the aggressiveness of established cancer (Kuroki et al, 1995). It has also been reported that loss of 4q is related to increased serum AFP (alpha-fetoprotein) levels (Yeh et al, 1996). In our CGH analysis of HCCs, the most commonly recurrent loss (4q) was observed in 45% of tumour specimens, the smallest region of overlap being 4q12–21. This result supported the view that chromosome 4q, particularly 4q12–21, harbours tumour suppressor activity that is lost during tumorigenesis in HCV-HCC.

We have explored possible correlations between clinicopathological characteristics and copy number changes in the tumours of our panel. The first objective of the statistical analysis was to examine relationship of total copy number changes with cancer stage. Information obtained from the univariate analysis (log-rank test) was applied to total copy number changes with cancer stage using the Cox model of proportional hazards, but we have found no relation between two factors. In the same way, we tried to analyse the relationship between copy number changes on specific chromosomes and tumour stage, but it could not be evaluated statistically because too few early-stage tumours (three cases) were among the total. Changes on 1p, 1q and 4q were detected in the HCCs classified as stage T1 (< 2 cm in diameter) with almost same frequency as more advanced HCCs.

The recurrently underrepresented sites observed in this CGH study, i.e. 1p, 5p, 16q and 17p, have also been reported to be common regions of allelic loss in HCC. Each of these segments is a known or suspected site of tumour suppressor genes, e.g. p53 at 17p13, *APC* at 5q21, *RB* at 13q14, *CDH1* at 16q21–24. Frequent genetic alterations in the distal region of chromosome 1p in HCCs suggest loss of this region is critical for initial hepatocarcinogenesis (Yeh et al, 1994). Recently p73, whose function is related to p53, was isolated from 1p36 (Kaghad et al, 1997). Unidentified suppressor genes associated with HCC appear to present on chromosome 8p (Emi et al, 1993; Fujiwara et al, 1994). The underrepresentation of 17p (45%) in our study is explained by loss of the p53 gene (17p13.1), a frequent feature of HCC (Fujimoto et al, 1994).

Over-representation of chromosome arm 1q was the most frequent feature in our series of tumours that shows copy-number increases; 16 of them exhibited gain involving 1q. Moreover, increased copy number at 1q has also been reported in a variety of other tumours including breast, gastric and neuroblastoma, all related to poor prognosis and sometimes to metastases (Borg et al, 1992; Tahara, 1995). Chromosome 1q contains the Abelson-related oncogene (*ARG*; Kruh et al, 1990) and the protein tyrosine phosphatase receptor type c polypeptide gene (*PTPRC*; Schaapveld et al, 1995), both of which are associated with cell growth and proliferation.

Overrepresentations of 17q and 8q, the second and third most common gains of DNA observed in this series, included amplification of 17q12 in one tumour. A potentially relevant gene on 17q12, *ERBB2*, encodes an EGF-like growth factor receptor (Kameda et al, 1990); another, whose product may contribute to control of cell proliferation and malignant transformation, is *MYC*; a gene that is over-expressed in most HCCs (Moroy et al, 1986). Gains at 6p (20% in our study) have not been previously described in HCC, although tumour-specific copy number increases at 6p have been reported in retinoblastomas and osteosarcomas (Cano et al, 1994; Forus et al, 1995), where *PIM1* may be one of the target genes.

CGH is particularly powerful in revealing discrete amplified loci. In our analysis, we detected four distinct amplification sites; 7q31, 11q13, 14q12 and 17q12. Putative target genes on 7q31, 11q13 and 17q12 are *MET*, *HST/INT2* and *ERBB2*, respectively, but amplification on 14q12 has never been reported in any other type of cancer. Therefore a novel gene whose overexpression is specific for HCC may exist there; amplification of this locus has

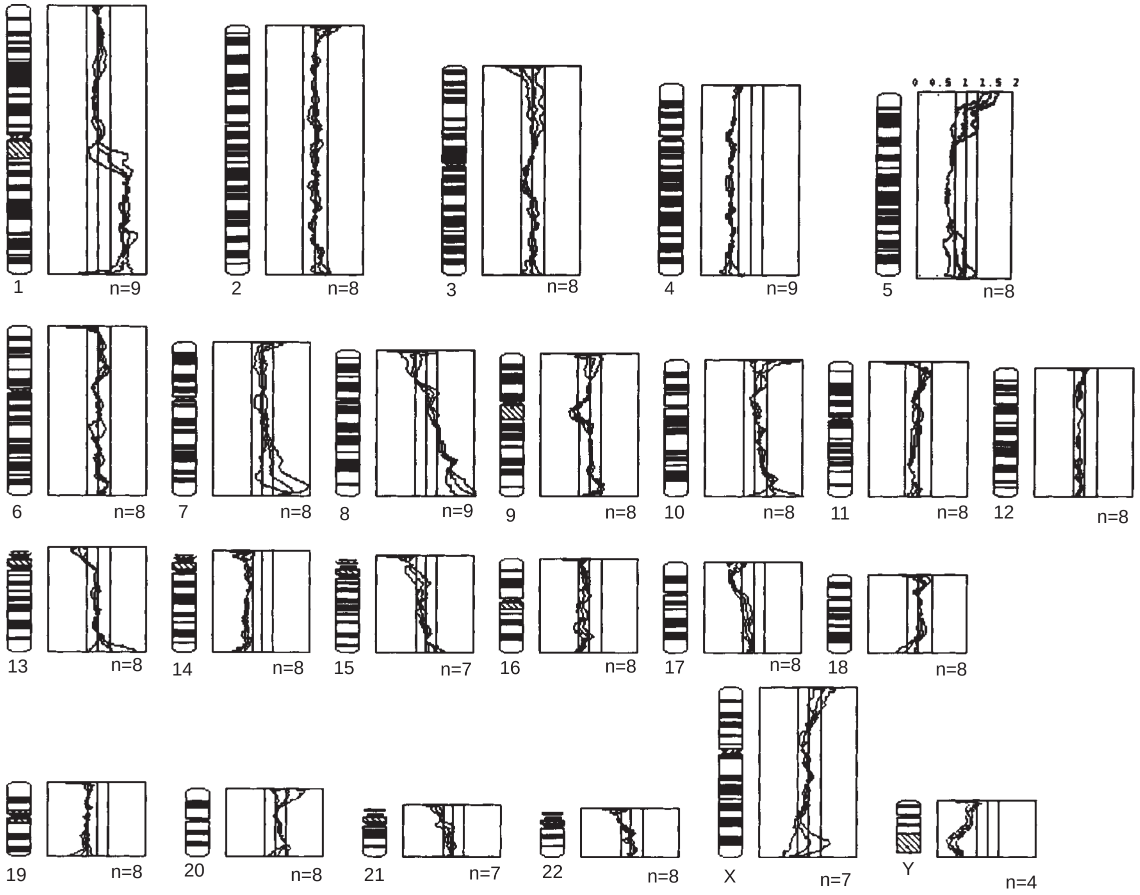

**Figure 2C** Computer-generated mean and standard deviation fluorescence ratio profiles for several metaphases from the hepatocellular carcinoma (Sample No 6). The central horizontal dashed line represents a green-to-red fluorescence ratio of 1 (no copy number change)

also been detected in HBV-HCC, along with amplifications of 11q13, 12p11 and 19q13 (Marchio et al, 1997). Amplifications we observed at 7q31, 11q13 and 17q12 have also been reported in other types of cancers (Tahara et al, 1994; Knuutila et al, 1998).

In summary, the recurrent copy number decreases we identified by CGH analysis support previous data on allelic loss in HCCs, and further implicate those sites as locations of tumour suppressor genes. Our study reveals that the pattern of chromosomal aberrations in HCC with HCV infection, although highly complex and involving virtually every chromosome, is clearly non-random and similar to CGH pattern of HCCs with HBV infection. In addition, many of the overrepresented chromosome segments observed in HCC are sites of known oncogene/growth-regulatory genes already implicated in tumorigenesis. Other overrepresented regions could be sites of novel amplified DNA sequences which may provide growth advantages in these aggressive neoplasms.

### ACKNOWLEDGEMENTS

This work was supported by grants-in-aid from the Ministry of Health and Welfare; the Ministry of Education, Science, Sports and Culture; the Organization for Pharmaceutical Safety and Research, Japan; a grant from Cell Fate Modulation Research Unit; and Research Grant of the Princess Takamatsu Cancer Research Fund.

### REFERENCES

Ariyama Y, Fukuda Y, Okuno Y, Seto M, Date K, Abe T, Nakamura Y and Inazawa J (1998) Amplification on double-minute chromosomes and partial-tandem duplication of the MLL gene in leukemic cells of a patient with acute myelogenous leukemia. *Genes Chromosomes Cancer* **23**: 267–272

Borg A, Zhang QX, Olsson H and Wenngren E (1992) Chromosome 1 alterations in breast cancer: allelic loss on 1p and 1q is related to lymphogenic metastases and poor prognosis. *Genes Chromosomes Cancer* **5**: 311–320

Buetow KH, Murray JC, Israel JL, London WT, Smith M, Kew M, Blanquet V, Brechot C, Redeker A and Govindarajah S (1989) Loss of heterozygosity suggests tumor suppressor gene responsible for primary hepatocellular carcinoma. *Proc Natl Acad Sci USA* **86**: 8852–8856

Cano J, Oliveros O and Yunis E (1994) Phenotype of variants malignancy, additional copies of 6p in retinoblastoma. *Cancer Genet Cytogenet* **76**: 112–115

Di Biscegli AM (1997) Hepatitis C and hepatocellular carcinoma. *Hepatology* **26**: 34S–38S

Emi M, Fujiwara Y, Ohata H, Tsuda H, Hirohashi S, Koike M, Miyaki M, Monden M and Nakamura Y (1993) Allelic loss at chromosome band 8p21.3–p22 is associated with progression of hepatocellular carcinoma. *Genes Chromosomes Cancer* **7**: 152–157

Fong TL, Shindo M, Feinstone SM, Hoofnagle JH and Di Bisceglie AM (1991) Detection of replicative intermediates of hepatitis C viral RNA in liver and serum of patients with chronic hepatitis C. *J Clin Invest* **88**: 1058–1060

Forus A, Weghuis DO, Smeets D, Fodstad O, Myklebost O and Geurts Van Kessel A (1995) Comparative genomic hybridization analysis of human sarcomas: II. Identification of novel amplicons at 6p and 17p in osteosarcomas. *Genes Chromosomes Cancer* **14**: 15–21

Fourel G (1994) Genetic and epigenetic alterations of gene expression in the course of hepatocarcinogenesis. In: *Liver Gene Expression*, Tronche F and Moshe Y (eds), pp. 298–343. R.G. Landes: Austin, TX

*© 1999 Cancer Research Campaign*

Fujimori M, Tokino T, Hino O, Kitagawa T, Imamura T, Okamoto E, Mitsunobu M, Ishikawa T, Nakagawa H, Hayada H, Yagura M, Matsubara K and Nakamura Y (1991) Allotype study of primary hepatocellular carcinoma. *Cancer Res* **51**: 89–93

Fujimoto Y, Hampton LL, Wirth PJ, Wang NJ, Xie JP and Thorgeirsson SS (1994) Alteration of tumor suppressor genes and allelic losses in human hepatocellular carcinomas in China. *Cancer Res* **54**: 281–285

Fujiwara Y, Ohata H, Emi M, Okui K, Koyama K, Tsuchiya E, Nakajima T, Monden M, Mori T, Kurimasa A, Oshimura M and Nakamura Y (1994) A 3-Mb physical map of the chromosome region 8p21.3–p22, including a 600-kb region commonly deleted in human hepatocellular carcinoma, colorectal cancer, and non-small cell lung cancer. *Genes Chromosomes Cancer* **10**: 7–14

Kaghad M, Bonnet H, Yang A, Creancier L, Biscan JC, Valent A, Minty A, Chalon P, Lelias JM, Dumont X, Ferrara P, McKeon, F and Caput D (1997) Monoallelically expressed gene related to p53 at 1p36, a region frequently deleted in neuroblastoma and other human cancers. *Cell* **90**: 809–819

Kallioniemi A, Kallioniemi OP, Sudar D, Rutovitz D, Gray JW, Waldman F and Pinkel D (1992) Comparative genomic hybridization for molecular cytogenetic analysis of solid tumors. *Science* **258**: 818–820

Kallioniemi A, Kallioniemi O-P, Piper J, Tanner M, Stokke T, Chen L, Smith HS, Pinkel D, Gray JW and Waldman FM (1994) Detection and mapping of amplified DNA sequences in breast cancer by comparative genomic hybridization. *Proc Natl Acad Sci USA* **91**: 2156–2160

Kameda T, Yasui W, Yoshida K, Tsujino T, Nakayama H, Ito M, Ito H and Tahara E (1990) Expression of ERBB2 in human gastric carcinomas; relationship between p185ERBB2 and the gene amplification. *Cancer Res* **15**: 8002–8009

Kasai Y, Takeda S and Takagi H (1996) Pathogenesis of hepatocellular carcinoma: a review from the viewpoint of molecular analysis. *Semin Surg Oncol* **12**: 155–159

Knuutila S, Bjorkqvist AM, Autio K, Tarkkanen M, Wolf M, Monni O, Szymanska J, Larramendy ML, Tapper J, Pere H, El-Rifai W, Hemmer S, Wasenius VM, Vidgren V and Zhu Y (1998) DNA copy number amplifications in human neoplasms: review of comparative genomic hybridization studies. *Am J Pathol* **152**: 1107–1123

Kruh GD, Perego R, Miki T and Aaronson SA (1990) The complete coding sequence of arg defines the Abelson subfamily of cytoplasmic tyrosine kinases. *Proc Natl Acad Sci USA* **87**: 5802–5806

Kuroki T, Fujiwara Y, Tsuchiya E, Nakamori S, Imaoka S, Kanematsu T and Nakamura Y (1995) Accumulation of genetic changes during development of and progression of hepatocellular carcinoma: Loss of heterozygosity on chromosome arm 1p occurs at early stage of hepatocarcinogenesis. *Genes Chromosomes Cancer* **13**: 163–167

Lowichik A, Schneider NR, Tonk V, Ansari MQ and Timmons CF (1996) Report of a complex karyotype in recurrent metastatic fibrolamellar hepatocellular carcinoma and a review of hepatocellular carcinoma cytogenetics. *Cancer Genet Cytogenet* **88**: 170–174

Marchio A, Meddeb M, Pineau P, Danglot G, Tiollais P, Bernheim A and Dejean A (1997) Recurrent chromosomal abnormalities in hepatocellular carcinoma detected by comparative genomic hybridization. *Genes Chromosomes Cancer* **18**: 59–65

Moroy T, Marchio A, Etiemble J, Trepo C, Tiollais P and Buendia MA (1986) Rearrangement and enhanced expression of c-myc in hepatocellular carcinoma of hepatitis virus infected woodchucks. *Nature* **324**: 276–279

Nagai H, Pineau P, Tiollais P, Buendia MA and Dejean A (1997) Comprehensive allelotyping of human hepatocellular carcinoma. *Oncogene* **14**: 2927–2933

Nalpas B, Driss F, Pol S, Hamelin B, Housset C, Brechot C and Berthelot P (1991) Association between HCV and HBV infection in hepatocellular carcinoma and alcoholic liver disease. *J Hepatol* **12**: 70–74

Nishida N, Fukuda Y, Ishizaki K and Nakao K (1997) Alteration of cell cycle-related genes in hepatocarcinogenesis. *Histol Histopathol* **12**: 1019–1025

Oda T, Tsuda H, Scarpa A, Sakamoto M and Hirahashi S (1992) P53 gene mutation spectrum in hepatocellular carcinoma. *Cancer Res* **52**: 6358–6364

Rogler CE and Chisari FV (1992) Cellular and molecular mechanisms of hepatocarcinogenesis. *Semin Liver Dis* **12**: 265–278

Sakakura C, Mori T, Sakabe T, Ariyama Y, Shinomiya T, Date K, Hagiwara A, Yamaguchi T, Takahashi T, Nakamura Y, Abe T and Inazawa J (1999) Gains, losses, and amplifications of genomic materials in primary gastric cancers analyzed by comparative genomic hybridization. *Genes Chromosomes Cancer* **24**: 299–305

Schaapveld RQ, Van Den Maagdenberg AM, Schepens JT, Weghuis DO, Geurts Van Kessel A, Wieringa B and Hendriks WJ (1995) The mouse gene Ptprf encoding the leukocyte common antigen-related molecule LAR: cloning, characterization, and chromosomal localization. *Genomics* **27**: 124–130

Simon D, Knowles BB and Weith A (1991) Abnormalities of chromosome 1 and loss of heterozygosity of 1p in primary hepatomas. *Oncogene* **6**: 765–770

Simon D and Carr BI (1995) Integration of hepatitis B virus and alteration of the 1p36 region found in cancerous tissue of primary hepatocellular carcinoma with viral replication evidenced only in noncancerous, cirrhotic tissue. *Hepatology* **22**: 1393–1398

Tabor E (1994) Tumor suppressor genes, growth factor genes, and oncogenes in hepatitis B virus-associated hepatocellular carcinoma. *J Med Virol* **42**: 357–365

Tahara E (1995) Molecular biology of gastric cancer. *World J Surg* **19**: 484–488

Takano S, Yokosuka O, Imazeki F, Tagawa M and Omata M (1995) Incidence of hepatocellular carcinoma in chronic hepatitis B and C: a prospective study of 251 patients. *Hepatology* **21**: 650–655

Tanaka K, Hirohata T, Koga S, Sugimachi K, Kanematsu T, Ohryohji F, Nawata H, Ishibashi H, Maeda Y, Kiyokawa H, Tokunaga K, Irita Y, Takeshita S, Arase Y and Nishino N (1991) Hepatitis C and hepatitis B in the etiology of hepatocellular carcinoma in the Japanese population. *Cancer Res* **51**: 2842–2847

The Liver Cancer Study Group of Japan (1994) Predictive factors for long term prognosis after partial hepatectomy for patients with hepatocellular carcinoma in Japan. *Cancer* **74**: 2772–2780

Tokino T, Fukushige S, Nakamura T, Nagaya T, Murotsu T, Shiga K, Aoki N and Matsubara K (1987) Chromosomal translocation and inverted duplication associated with integrated hepatitis B virus DNA in hepatocellular carcinomas. *J Virol* **61**: 3848–3854

Tsuda H, Zhang W, Shimosato Y, Yokota J, Terada M, Sugimura T, Miyamura T and Hirohashi S (1990) Allele loss on chromosome 16 associated with progression of human hepatocellular carcinoma. *Proc Natl Acad Sci USA* **87**: 6791–6794

Van Rensburg SJ, Cook-Mozafarri P, Van Schlkwyk DJ, Van Der Watt JJ, Vincent TJ and Purchase IF (1985) Hepatocellular carcinoma and dietary aflatoxin in Mozambique and Transkei. *Br J Cancer* **51**: 713–726

Wang J, Chenivesse X, Henglein B and Brechot C (1990) Hepatitis B virus integration in a cyclin A gene in a hepatocellular carcinoma. *Nature* **343**: 555–557

Whelan SL, Parkin DM and Masuyer E (eds) (1993) *Trends in Cancer Incidence and Mortality*. IARC Scientific Publ., No. 102. IARC: Lyon

Yeh SH, Chen PJ, Chen HL, Lai MY, Wang CC and Chen DS (1994) Frequent genetic alterations at the diastal region of chromosome 1p in human hepatocellular carcinomas. *Cancer Res* **54**: 4188–4192

Yeh SH, Chen PJ, Lai MY and Chen DS (1996) Allelic loss on chromosome 4q and 16q in hepatocellular carcinoma: association with elevated α-fetoprotein production. *Gastroenterology* **110**: 184–192

Zhang W, Hirohashi S, Tsuda H, Shimosato Y, Yokota J, Terada M and Sugimura T (1990) Frequent loss of heterozygosity of chromosomes 16 and 4 in human hepatocellular carcinoma. *Jpn J Cancer Res* **81**: 108–111

*© 1999 Cancer Research Campaign*
